# Beyond sport to combat childhood obesity: educating, engaging, and preventing through an integrated school-based campaign grounded in the values of Olympism

**DOI:** 10.3389/fendo.2025.1636728

**Published:** 2025-07-09

**Authors:** Gianvincenzo Zuccotti, Franco Bruno Ascani, Assunta Ferdinanda Romeo, Valeria Calcaterra, PODiaCar Team

**Affiliations:** ^1^ Pediatric Department, Buzzi Children’s Hospital, Milano, Italy; ^2^ Department of Biomedical and Clinical Sciences, University of Milan, Milan, Italy; ^3^ International Federation of Sport Cinema and Television, Milano, Italy; ^4^ Department of Internal Medicine and Therapeutics, University of Pavia, Pavia, Italy

**Keywords:** childhood obesity, children, PODiaCar, Trofei di Milano Cortina, Olympism, sport

## Abstract

**Introduction:**

Promoting sports in schools is a key strategy to address childhood obesity and raise awareness of the benefits of physical activity. The “Trofei di Milano Cortina 2026” initiative, in collaboration with Buzzi Children’s Hospital in Milan, launched a comprehensive school-based campaign to promote Olympism and healthy lifestyles.

**Methods:**

The initiative involved 350 schools across primary and lower secondary levels, offering free educational and sports activities in two phases: a year-long educational component followed by a final sports event. Educational materials, including an animated cartoon “Grandma Wilma’s Tales,” supported the EU-backed project on combating pediatric obesity and educating children on diabetes and cardiovascular risk.

**Results:**

Out of the targeted schools, 95 (27.1%) formally joined, involving 2.100 classes and 51.066 students aged 6 to 13. Participation in educational activities was substantial, with thousands of modules completed across five thematic areas, such as “Sport in a Video,” “Sustainability Champion,” and “Education 4.0.” The initiative engaged 312 teachers and included hospital school participation. The sporting phase registered 8,658 participants, nearly evenly split by gender. A network of 10 public and sports institutions, along with two sponsors, supported the program, highlighting the initiative’s wide-reaching impact and collaborative strength in fostering youth health and well-being.

**Conclusions:**

The data confirm the initiative’s success, highlighting strong community engagement and broad acceptance of the educational model. Integrating sport, values, and education proves to be an effective, scalable strategy for positively and collectively preventing childhood obesity.

## 1 Introduction

Promoting healthy lifestyles through physical activity and health education is a key objective of the World Health Organization (WHO) (1). Regular physical activity plays a crucial role in both the prevention and management of noncommunicable diseases (NCDs), including cardiovascular diseases, stroke, diabetes, and various forms of cancer (2–5). Among children and adolescents, physical activity also supports bone health, fosters healthy growth and muscle development, and enhances motor skills as well as cognitive function (6). Moreover, it contributes to the regulation of energy expenditure, which is essential for maintaining energy balance, managing body weight, and preventing obesity (7–10).

Despite the well-documented benefits of physical activity, global levels remain alarmingly low. Approximately 81% of adolescents aged 11 to 17 do not meet the WHO’s recommended levels of physical activity. This trend is especially pronounced among girls, with 85% falling short of guidelines, compared to 78% of boys (11).

Unhealthy lifestyle behaviors are strongly associated with the increasing prevalence of pediatric obesity (12–14). Although the pathogenesis of obesity is multifactorial, encompassing genetic, epigenetic, and environmental factors, poor dietary habits and physical inactivity and sedentary behavior play a significant role in its development and constitute one of the elements of its prevention (15, 16). Physical activity levels are influenced by a wide range of factors, including individual behaviors and broader social, cultural, environmental, and economic conditions (17, 18).

In this context, schools play a pivotal role in the holistic development of children, intellectually, physically, and socially. In today’s society, increasingly marked by sedentary behavior and excessive screen time, the responsibility of educational institutions to foster an active and healthy lifestyle has become more critical than ever. Through a combination of educational and recreational opportunities, alongside the promotion of healthy daily routines, schools can significantly contribute to raising health-conscious, energetic, and well-rounded individuals (19).

Instilling the value of physical activity and sport from an early age helps lay a strong foundation for lifelong physical, mental, and emotional well-being. As highlighted by the International Olympic Committee (20), sport is recognized as a powerful educational tool, offering a universal language to convey essential messages on healthy lifestyles, social inclusion, gender equality, and community rebuilding. Moreover, embedding physical education within the core values of Olympism, such as excellence, friendship, and respect, can play a transformative role in addressing the stigma often associated with obesity (20). By emphasizing respect for oneself and others, promoting inclusion, and celebrating personal progress rather than competition alone, these values foster a supportive environment where children of all body types feel valued and empowered. Enhancing sports activities within the school setting could represents a crucial investment, not only for combating childhood obesity but also for deepening education on the multifaceted benefits of physical activity (21, 22).

In this report, we present an integrated, school-based model for promoting sports and the values of Olympism as a means of protecting children’s health. As part of this model, the historic initiative “Trofei di Milano Cortina 2026,” which promotes physical activity in schools, has, for its 62th edition, joined forces with the Buzzi Children’s Hospital in Milan (Italy) to launch an educational and awareness campaign aimed at combating childhood obesity. The integration of health initiative into public school, may be effective in promoting healthier lifestyle habits among children from an early age.

## 2 Methods

### 2.1 Synergic education programs

The proposal involved the synergistic collaboration between the “Trofei di Milano Cortina 2026” project and the EU-supported project “Combating Pediatric Obesity: From a Predictive Tool for Type 2 Diabetes and Cardiovascular Risk to Health Education Programs” (PODiaCar, EU4H-2022-PJ-3 — EU4H-PJG, Project number 101128946).

“Trofei di Milano Cortina 2026” is part of the Gen26 Education Program that is a project launched by The Italian National Olympic Committee to inspire children to take up sport in the build up to the Milan Cortina 2026 Winter Olympic and Paralympic Games. “Trofei di Milano Cortina 2026” is aimed at primary and lower secondary school students in the Lombardy and Veneto regions (Italy). The goal is to bring young people closer to Olympism and its values, such as excellence, friendship, respect.

PODiaCar project is an initiative led by Buzzi Children’s Hospital in Milan, Italy, in partnership with the University of Pavia (Italy), the University of Granada (Spain), LUNEX University of Applied Sciences (Luxembourg), and ASOMI College of Science (Malta). The initiative seeks to combat childhood obesity and its associated complications, such as type 2 diabetes and cardiovascular diseases. By harnessing multidisciplinary collaboration and advanced digital technologies, including an educational cartoon, artificial intelligence, and digital twins, PODiaCar promotes a proactive, data-driven strategy for improving child health and preventing disease. PODiaCar supports the European Commission’s ‘Healthier Together’ campaign targeting major NCDs and aligns with the strategic objectives of the European Health and Digital Executive Agency (HaDEA).

### 2.2 Program activities

As part of the project initiative, 350 schools (1st and 2nd grades of primary school and primary and lower secondary schools) were engaged through an initial email that provided key information about the program, including its educational value, participation procedures, operational materials, and the proposed schedule of activities. This communication was sent both to the schools’ official institutional addresses and to the physical education coordinators. Subsequently, the official regulations and participation form were also distributed. At a later stage, thanks to the collaboration with the Regional School Office for Lombardy, these documents were published on the Office’s official website and shared with all schools in the region through an institutional communication.

All activities are free of charge, involve teachers, and are structured in two phases: an educational phase throughout the school year and a sporting phase that culminates in the finals, traditionally held at the Gianni Brera Arena in Milan (Italy).

The educational activities are organized into five operational modules, each representing a distinct thematic pathway and identified by a specific color (ORANGE, Your “Sport in a Video; BLUE, Your “Lifestyles; GREEN, Sustainability Champion; YELLOW, Europe of the games; PINK, Education 4.0, see **Table 1**).

**Table 1:** Operational modules of the proposed educational activities.

Module	Thematic pathway	Description
ORANGE	Your Sport in a Video	Students are invited to transform themselves into filmmakers and actors as they explore one of these extraordinary stories. Together with their classmates and Teachers, they will be able to create and interpret a video (lasting no more than 4 minutes) that tells the story of an Olympic or Paralympic athlete, or describes an iconic moment in the history of the Games that particularly affected and inspired them. Students will have the opportunity to make a video in which they themselves will be the protagonists, playing the athletes or historical figures who made the Games unforgettable.
BLUE	Your Lifestyles	Students are invited to create and design the perfect healthy dish to be eaten before an Olympic or paralympic race, including the elements for a balanced meal for a sportsman with: carbohydrates, protein, healthy fats, fruits and vegetables. Students can have fun writing their own personal recipe and their own dish remembering that eating healthy is essential to feeling good and performing at one’s best in sports.
GREEN	Sustainability Champion	Students are invited to create a slogan, accompanied by a drawing or video, that clearly and creatively expresses the daily actions each of us can take to safeguard the planet by turning each student into a champion of sustainability, just like their Sports Heroes.
YELLOW	Europe of the games	Each student can participate by drawing and telling stories: - A European city, which has hosted or will host the Games. The drawing can include: famous monuments of the chosen city, the Olympic stadium, sports facilities, etc. - An athlete starring in that edition and representing the values of the European Union mentioned above, motivating the choice - European Union value behind the choice
PINK	Education 4.0	Students are invited to tell, with a text/slogan accompanied by a drawing and/or video, how they use technology properly or avoid becoming addicted to it. The goal is to show how they can use their free time creatively, such as playing sports or rediscovering traditional games, and to understand the risks of using excessive use of technology.

The sporting phase was specifically planned for participation in sports activities, including SPRINT RUN (60 m – 80 m) and FantAthletics (200 m), which represent the key activities of the final event.

Throughout the school year, the children participated in physical education activities as outlined in the national curriculum, amounting to 2 hours per week. These activities included various games with balls, frisbees, and jump ropes, as well as group games for the 1st and 2nd grades of primary school. In both primary and lower secondary schools, students engaged in more structured activities and traditional sports such as volleyball, basketball, and soccer. All initiatives helped prepare the students for the final event and were designed to encourage active student involvement, promote interdisciplinarity, and foster participation.

During the educational phase, the EU-supported PODiaCar project was promoted and disseminated. Specifically, an educational animated cartoon titled ‘Grandma Wilma’s Tales,’ created as part of the project to promote healthy habits among young children and their families, along with educational materials on healthy lifestyles, was distributed in the schools participating in the ‘Trofei di Milano Cortina 2026’ initiative.

The planning, implementation, and closing phases of the school activities took place from 15 September 2024 to 9 May 2025.

### 2.3 Network of collaborators

All activities were coordinated by Fondazione Milano Cortina 2026, responsible for organizing the 2026 Winter Olympic and Paralympic Games, established by the Italian National Olympic Committee, the Italian Paralympic Committee, the Lombardy and Veneto Regions, the Autonomous Provinces of Trento and Bolzano, and the Municipalities of Milan and Cortina d’Ampezzo; the International Federation of Sport Cinema and Television (FICTS), which coordinates the film and audiovisual activities related to the initiative; and the Italian Association for Culture and Sport (AICS), managing the technical and logistical organization of competitions and activities. Additionally, the network of collaborators invited to join the initiative included institutional bodies, sports and cultural associations, as well as private sponsors, whose support helped cover the event’s costs and broaden its impact. The program was supported by European Commission- Representation in Italy.

The integrated campaign to combat childhood obesity, conducted between Buzzi Children’s Hospital and participating schools, was carried out in accordance with the guidelines of the Declaration of Helsinki and approved by the Institutional Review Board Territorial Lombardia 1 (protocol number CET 202-2023). All participants, or their legal guardians, provided written informed consent after being fully informed about the nature of the study.

## 3 Statistical analysis

Quantitative data were presented as mean ± SD or median with interquartile range, and qualitative data as counts and percentages. We tested for normality by Shapiro–Wilk tests and graphically checked for linearity. Statistical analyses were performed using Student’s t-test and chi-square test. All statistical analysis was performed using stata v.18.0 stataCorp USA software.

## 4 Results

A total of 95 schools (27.1%) formally joined the initiative (57 from Milan and its province in the Lombardy region, and 32 from Treviso, Belluno, and Verona and their provinces in the Veneto region) representing 2.100 classes. This partial participation reflects several contextual challenges. Many schools expressed interest but were ultimately unable to formally join due to administrative burdens or resource limitations. Additionally, participation required alignment with curricular priorities, which was more feasible for some schools than others. The hospital school also participated in the initiative with a single class.

The total number of children involved was 51.066, with an age range between 6 and 13 years. Gender distribution was balanced: 48.8% boys and 51.2% girls (p>0.05).

### 4.1 Educational phase

The educational activities, divided into different areas, saw significant participation with a high number of completed modules, specifically: 5,523 ORANGE modules Your “Sport in a Video with 2,921 self-produced videos (average duration 60 seconds) by the students; 5,745 YELLOW modules “Europe of the games”; 5,395 GREEN modules “Sustainability Champion”; 5,270 BLUE modules “Your Lifestyles”; and 5,344 PINK modules “Education 4.0”.

The educational cartoon “Grandma Wilma’s Tales” was shown in all schools (the cartoon can be viewed at the link https://drive.google.com/file/d/1xK25uA8KU53AQ8enDTtD_Ey0c2jc_0i6/view?usp=share_link). The story follows a boy named Vitto and his dog Buzz as they learn about the risks of poor diet and inactivity, and the benefits of a balanced, healthy lifestyle. Using simple language, clear visuals, and relatable characters, the cartoon encourages children to make healthier choices daily. The friendship and respect between Vitto and Buzz are key to maintaining good health.

The distribution of materials aimed at introducing children to a healthy lifestyle (see Additional File 1) was well received by all participating classes and actively used by teachers. Teacher engagement was significant, with a total of 312 educators actively involved in the proposed activities.

### 4.2 Sporting phase

A key feature of the final event was the organization of sports competitions, divided by age group and school level sprint races of 60 m and 80 m (qualifiers, semifinals, and finals) for athletes from primary and lower secondary schools and FantAthletics (200 m for athletes in the 1st and 2nd grades of primary school). A total of 8,658 participants registered for the sports activities (49.7% male and 50.3% female). During the final event, the awards ceremony took place for the initiatives of the educational phase and the sports competitions. The final event was attended by 35 local and regional delegations, partner organizations, sponsors, Olympic and Paralympic athletes, school principals, teacher coordinators, and a large number of parents, with a total of around 11,000 spectators.

### 4.3 Collaborative network

In collaboration with Fondazione Milano Cortina 2026, FICTS, and the Italian Association for Culture and Sport, a network of 10 collaborators, including local, regional, and national institutions, as well as local federations, and 2 sponsors joined the initiative.

The overall organization of the initiative required the operational support of 83 people, including trainers, technicians, coordinators, volunteers, and logistics staff.

The total estimated cost for the implementation of the project was approximately 90.000 euros, covering materials, venues, professional fees, and communication.

## 5 Discussion

The data collected confirm the success of the initiative, demonstrating strong community involvement and the effective acceptance of the proposed educational model. The combination of sport, values, and education is a concrete, scalable, and culturally powerful strategy to address obesity in a preventive, positive, and collective way. The Olympic Games, alongside other major international initiatives, represent an outstanding opportunity to transform this vision into impactful and lasting action.

Obesity is a complex condition driven by the interaction of biological, genetic, social, environmental, and behavioral factors; therefore, effectively addressing childhood obesity demands multifaceted strategies that comprehensively target these determinants of health (23–26). Promoting regular physical activity from an early age can play a key role in reducing the risk of obesity and supporting overall well-being. Our program advocates for a comprehensive, school-based intervention model that synergizes educational, engagement, and preventive components, anchored in the foundational values of Olympism.

As highlighted by Pulimeno et al. (27), schools, as primary educational institutions, should embed health promotion into everyday teaching and learning practices, embracing the principle of “better health through better schools”.

With physical activity levels declining significantly among children, the role of schools, where children spend a large portion of their day, has become increasingly crucial for effective health interventions (28). As confirmed by the high level of school adherence to our program and children’s participation, schools serve as essential environments for health promotion, offering structured opportunities not only for physical activity but also for social development during these formative years.

To maximize their impact on obesity prevention and the promotion of physical activity and sport, school-based interventions must prioritize the content, quality, duration, and consistency of physical activity delivered (28). We established a collaborative synergy between sports organizations and healthcare institutions, serving as a bridge between health promotion and active participation, resulting in a continuous educational campaign throughout the entire school year. This campaign was enriched with structured content designed within a comprehensive framework aimed at promoting children’s health.

The integrated, multidisciplinary approach, involving educators, healthcare professionals, families, and community stakeholders, enhances the intervention’s reach and sustainability. Additionally, the adoption of innovative pedagogical tools, as animated cartoon, aligned with these values may increase engagement and efficacy in health education programs (29).

Embedding the campaign within the values of Olympism, as excellence, friendship and respect (20), introduces an ethical and psychosocial dimension essential to mitigating obesity-related stigma (30, 31). Weight stigma is widespread during childhood and adolescence and can affect individuals throughout their lives (32). It often leads to negative consequences such as low self-esteem, depression, anxiety, social isolation, and can contribute to unhealthy eating behaviors and weight gain, ultimately impacting long-term physical and mental health (33, 34). As reported by Bevan et al. (35), the initiatives seeking to increase participation in physical initiative and sport may need to address weight stigma and associated appearance related concerns. The Olympism principles, also included in the animated cartoon, facilitate an inclusive and supportive environment that fosters positive self-identity and social cohesion, factors known to enhance adherence to healthy behaviors among children with diverse body types.

Finally, capitalizing on the global influence of the Olympic movement and analogous international initiatives can potentiate widespread cultural change, promoting health equity and collective responsibility (36, 37). This multidimensional strategy underscores the potential for scalable, culturally congruent interventions to reduce the prevalence of childhood obesity and its associated comorbidities.

Although no formal satisfaction or response questionnaires were collected, which may represent a limitation in assessing the perceived impact of the campaign, the high level of participation and continued engagement throughout the project can be considered an indirect but meaningful indicator of its positive reception and relevance. For future editions, a survey will be considered to better understand the children’s level of appreciation in accordance with their age.

Additionally, we do not have weight-related data for the children involved in the project, as the study design did not include anthropometric measurements. However, recognizing the added value such data could offer in evaluating the effectiveness of the project, we would consider including them in future evaluations.

## 6 Conclusion

The data collected support the success of an integrated school-based campaign to engage the community and raise awareness about the promotion of healthy lifestyles. The combination of sport, the values associated with it, and education on healthy living represents a concrete and viable strategy to promote health, combat pediatric obesity, and move forward toward the future with principles of social inclusion and integration. Future policies must support the expansion of integrated sport and health education initiatives within schools, ensuring equitable opportunities for all children to develop healthy habits and reduce obesity rates.

## Data Availability

The original contributions presented in the study are included in the article/supplementary material. Further inquiries can be directed to the corresponding author.

## Group members of PODiaCar Team

ITALY Elvira Verduci (Buzzi Children’s Hospital and University of Milano, Milano, Italy), Savina Mannarino (Buzzi Children’s Hospital and University of Milano, Milano, Italy), Virginia Rossi (Buzzi Children’s Hospital and University of Milano, Milano, Italy), Giulia Fiore (Buzzi Children’s Hospital and University of Milano, Milano, Italy), Martina Tosi (Buzzi Children’s Hospital and University of Milano, Milano, Italy), Lucia Labati (Buzzi Children’s Hospital and University of Milano, Milano, Italy), Matteo Vandoni (University of Pavia, Italy), Luca Marin (University of Pavia, Italy), Vittoria Carnevale Pellino (University of Pavia, Italy), Alessandro Gatti (University of Pavia, Italy), Caterina Cavallo (University of Pavia, Italy), Stefano Conca Bonizzoni (Officine Creative, University of Pavia, Italy); SPAIN Cristina Campoy (University of Granada, Granada, Spain), Mireia Escudero Marin (University of Granada, Granada, Spain), Rocio Bonillo Leon (University of Granada, Granada, Spain), Mercedes García-Bermúdez (University of Granada, Granada, Spain), Antonio E. Jerez-Calero (University of Granada, Granada, Spain), Inmaculada Guerrero, Ricardo Pérez Iañez (University of Granada, Granada, Spain), José M^a^ Gómez Vida (University of Granada, Granada, Spain); LUXEMBOURG Camilo Corbellini (University of Applied Sciences, Differdange, Luxembourg), Raphael Martins de Abreu (University of Applied Sciences, Differdange, Luxembourg), Khatija Bhadur (University of Applied Sciences, Differdange, Luxembourg); MALTA Valter Pagani (Asomi College of Malta, Malta), Umberto Ciriello (Asomi College of Malta, Malta), Francesco Conti (Asomi College of Malta, Malta).
